# Supplementary material for: Improved selection of canonical proteins for reference proteomes
Source: NAR Genom Bioinform. 2024 Jun 8;6(2):lqae066. doi: 10.1093/nargab/lqae066 (PMC11165316; doi:10.1093/nargab/lqae066)
Supplement: lqae066_Supplemental_File [file lqae066_supplemental_file.pdf]

SUPPLEMENTAL FIGURES

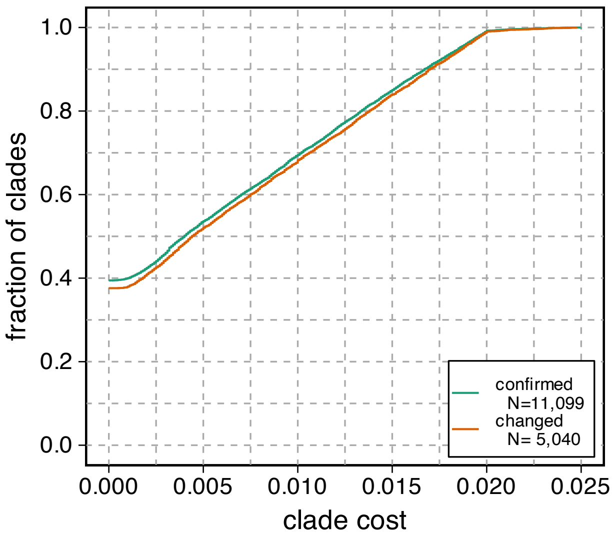

Suppl. Fig. 1: Distribution costs in clades with confirmed or proposed changes in the canonical isoform assignment. The fraction of all confirmed or proposed clades is plotted against the gap-based clade cost. Clades with zero cost contain sequences that align without gaps from beginning to end.

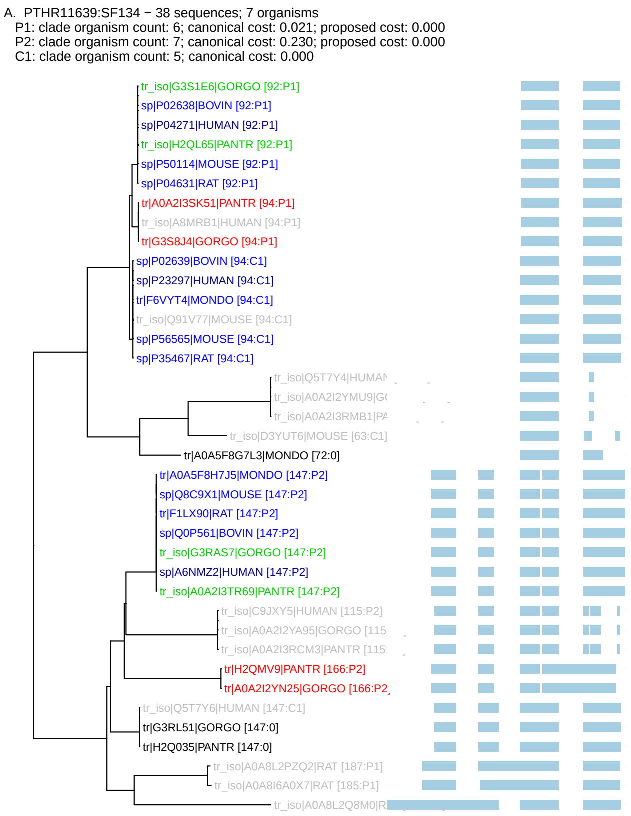

Suppl. Fig. 2: A phenogram showing three clades found for members of the PTHR11639:SF134 ortholog group, that contains S100-A1 small calcium binding proteins. Sequences are labeled as in Fig. 3, but here there are two clades with proposed changes (`P1` and `P2`), as well as a confirmed clade (`C1`).

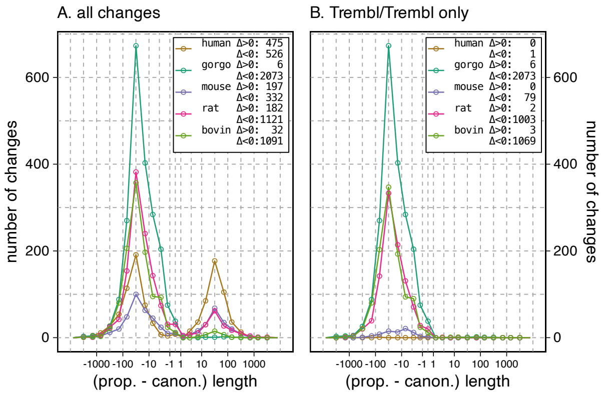

Suppl. Fig. 3: Changes in canonical sequence length for proposed changes. Differences between the proposed and current canonical sequence lengths for five different proteomes are shown. (A) All proposed canonical changes, including SwissProt to SwissProt, SwissProt to TrEMBL, and TrEMBL to TrEMBL; (B) Only TrEMBL to TrEMBL changes. Numbers in the legend box indicate the total number of changes proposed for that organism. Only 11 of the 4,238 proposed Trembl/Trembl changes increase the length of the canonical sequence for these 5 proteomes.

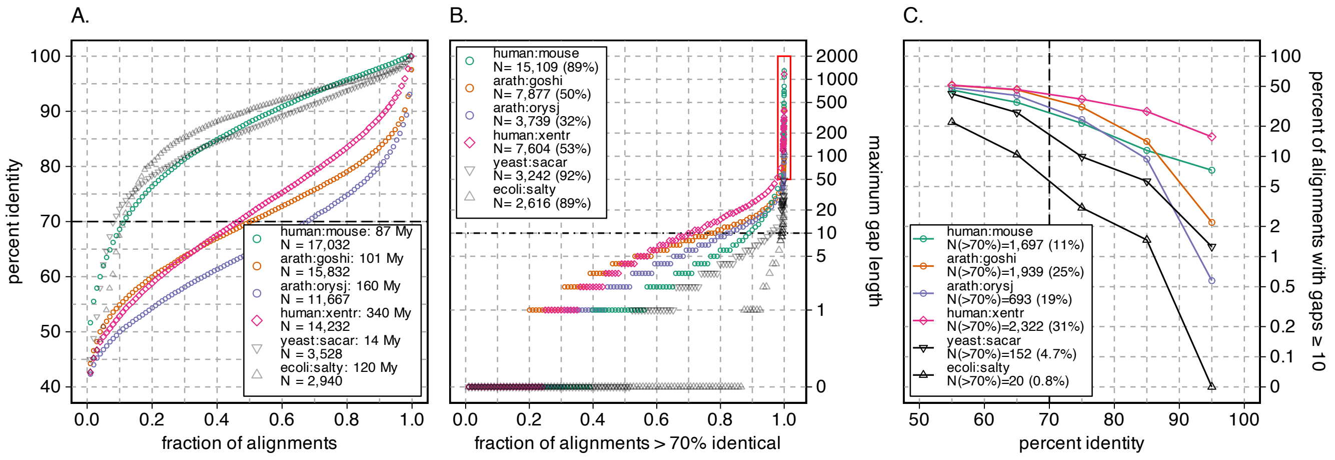

Suppl. Fig. 4: Comparison of sequence identity and gap lengths in more divergent proteins. This figure replots the data from Fig. 1 for `human:mouse`, `yeast:sacar`, and `ecoli:salty`, and adds identity and gap distribution data for three more distant pairs of organisms, human vs *X. tropicalis* (`human:xentr`, 340 My), and two plant pairs: *A. thaliana* vs cotton (`arath:goshi`, 101 My) and rice (`arath:orysj`, 160 My). Panel (A) shows the identity distributions; (B) the maximum gap lengths in alignments that are more than 70% identical, and (C) the number (and fraction) of sequences $>$70% identical with gaps $\geq$10.
